# *Paraburkholderia phytofirmans* PsJN delays *Botrytis cinerea* development on grapevine inflorescences

**DOI:** 10.3389/fmicb.2022.1030982

**Published:** 2022-10-20

**Authors:** Lidiane Carla Miotto Vilanova, Marine Rondeau, Mathilde Robineau, Jean François Guise, Céline Lavire, Ludovic Vial, Florence Fontaine, Christophe Clément, Cédric Jacquard, Qassim Esmaeel, Essaïd Aït Barka, Lisa Sanchez

**Affiliations:** ^1^Université de Reims Champagne-Ardenne, RIBP USC INRAE 1488, Reims, France; ^2^Université Claude Bernard Lyon 1, CNRS, INRAE, VetAgro Sup, UMR Ecologie Microbienne, Villeurbanne, France

**Keywords:** *Botrytis cinerea*, protection, grapevine inflorescences, antigerminative effect, *Paraburkholderia phytofirmans* PsJN

## Abstract

Grapevine flowering is an important stage in the epidemiology of *Botrytis cinerea*, the causal agent of gray mold disease. To prevent infection and to minimize postharvest losses, the control of this necrotrophic fungus is mainly based on chemical fungicides application. However, there is a growing interest in other control alternatives. Among them, the use of beneficial microorganisms appears as an eco-friendly strategy. This study aims to investigate the effect of *Paraburkholderia phytofirmans* PsJN, root-inoculated or directly sprayed on fruiting cuttings inflorescences to control *B. cinerea* growth. For this purpose, quantification by real time PCR of *Botrytis* development, direct effect of PsJN on fungal spore germination and chemotaxis were assayed. Our results showed a significant protective effect of PsJN only by direct spraying on inflorescences. Moreover, we demonstrated an inhibition exerted by PsJN on *Botrytis* spore germination, effective when there was a direct contact between the two microorganisms. This study showed that PsJN is positively attracted by the pathogenic fungus *B. cinerea* and forms a biofilm around the fungal hyphae in liquid co-culture. Finally, microscopic observations on fruit cuttings revealed a co-localization of both beneficial and pathogenic microorganisms on grapevine receptacle and stigma that might be correlated with the protective effect induced by PsJN against *B. cinerea via* a direct antimicrobial effect. Taking together, our findings allowed us to propose PsJN as a biofungicide to control grapevine gray mold disease.

## Introduction

Grapevine, one of the most important fruit cultivated worldwide, is affected by diverse pre- and post-harvest pathogens that entail drastic production losses. Among these pathogens, *Botrytis cinerea*, a widespread phytopathogenic fungus causing the gray mold disease affects the leaf stems, flowers, and fruits of plants under wet conditions and favorable temperatures (Williamson et al., 2007; Oliveira et al., 2009). This necrotrophic fungus, considered as the second most dangerous phytopathogen worldwide (Dean et al., 2012), leads to significant economic damages to viticulture. Flowering is the first opportunity for latent infection but it may occur at any time thereafter (Keller et al., 2003; Viret et al., 2004; Hahn, 2014). Indeed, *B. cinerea* can attack developing inflorescences, causing in severe cases the drying out and dropping of the whole inflorescences. *Botrytis* can be established on an area between the calyx and the base of the stigma and remains in latency until maturation of the berries (Viret et al., 2004; Ciliberti et al., 2015). Before flowering, the flower buds are covered with a cap, or calyptre, which is detached from the base of the ovary and then falls. When the weather conditions are wet during bloom and depending on the grape varieties, flower hoods and stamens can remain attached to the peduncle of the berry. These dehiscent tissues are especially conducive to the development of *B. cinerea*. In addition, flowers are highly susceptible to *B. cinerea* infection due to the low resveratrol content (Keller et al., 2003) and pollen abundance (Lehoczky, 1975). Thus, the flowers infection by *B. cinerea* appears to be a key stage in the epidemiological development of the fungus on grapevine. A model to study the development of inflorescences under controlled conditions has been developed: the fruiting cuttings produce flowering comparable to flowering in the vineyard (Lebon et al., 2005).

Control of *B. cinerea* is mainly based on the application of chemical fungicides to prevent infection and to minimize postharvest losses (González-Domínguez et al., 2018). However, these practices have adverse effects on the environment and on the health of users and promote the development of resistant strains (Hahn, 2014). Therefore, there is a growing interest in other control alternatives and among them, the use of beneficial microorganisms appears as an eco-friendly strategy. Among these, plant growth promoting rhizobacteria are of great interest (Lugtenberg and Kamilova, 2009) since they induce not only stimulation of plant growth but can also protect against pathogens (Beneduzi et al., 2012). Different studies described biological control agents (BCAs) that protect grapevine against gray mold disease (Abbey et al., 2019; Bruisson et al., 2019; Esmaeel et al., 2019, 2020; Amarouchi et al., 2021; Librizzi et al., 2022). Previous work on grapevine *in vitro*-plantlets demonstrated that *Paraburkholderia phytofirmans* strain PsJN can protect leaves against *Botrytis* by a combined effect: defense priming and direct antifungal effect (Miotto-Vilanova et al., 2016). Moreover, Compant et al. (2008) showed that the strain PsJN is able to migrate until inflorescences stalks in fruit cuttings. The present work aimed to investigate the ability of PsJN to protect grapevine fruiting cuttings against *B. cinerea* after different modes of application: root-inoculation vs. direct spraying on inflorescences and to deeper investigate the direct bacterium-fungus interaction.

## Materials and methods

### Microorganisms and growth conditions

*Paraburkholderia phytofirmans* strain PsJN-*gfp* was cultivated as described in Miotto-Vilanova et al. (2019). Inoculation of plants by *P. phytofirmans* was carried out at a bacterial concentration of 10^6^ CFU ml^−1^. *Botrytis cinerea* strain 630 (Bc630) was originally taken from the Champagne vineyards (INRA, Versailles, France). Liquid cultures of *B. cinerea* were prepared from stock cultures stored in glycerol at −80°C. For culture, 25 ml of PDB (Potato Dextrose Broth, 12 g L^−1^) were seeded with 250 μl of stock before to be cultured for 7 days at 20°C and 140 rpm with a photoperiod of 16/8 h (80 μmol m^−2^ s^−1^). This pre-culture was then crushed and spread on Petri dishes containing PDA. The seeded Petri dishes were cultured for 3 weeks at 20°C to produce conidia. For the infection, suspensions of conidia (10^3^ conidia ml^−1^) were prepared in PDB medium. These suspensions were placed at 20°C and 150 rpm for 3 h to initiate the germination of the conidia. Carbon source utilization of *P. phytofirmans* PsJN was measured as described in Nguyen-Huu et al. (2022).

### Plant material and growth conditions

Three-node cuttings (20 cm long) of *Vitis vinifera* L. cv. Pinot noir were cane pruned from Champagne vineyard (France). Fruiting cuttings were then prepared according to Petit et al. (2009). Cuttings were then stored in the dark at 4°C for 6–8 weeks. Before use and after 15 h of hydration at 28°C, the two proximal nodes were removed, and the cuttings were immersed for 15 s in indole-3-butyric acid solution (1 g L^−1^) to promote rhizogenesis. The cuttings were then placed on plastic plates filled with a mixture of soil/perlite/sand (4/1/1). The plates were placed on a 300w Puteaux^®^ heating blanket (25°C) in a cold chamber (8°C) to promote the emergence of roots, without buds discharging. Four weeks after, each cutting was then placed in a plastic pot (9 cm × 9 cm × 10 cm) filled with 250 g of potting soil (special Gramoflor, Gramoflor GmbH & Co. KG, Vechta, Germany). The potted cuttings were irrigated daily with tap water and incubated in a growth chamber at 25°C/20°C day/night temperature, 16 h photoperiod and 70% relative humidity. To avoid the beginning of vegetative growth and to facilitate development of inflorescence, leaves were removed daily according to Lebon et al. (2005).

### Effect of *Paraburkholderia phytofirmans* PsJN on *Botrytis cinerea* spore germination

The effect of PsJN on spore germination was carried out according to the procedure described previously (Miotto-Vilanova et al., 2016). In addition, the non-contact direct effect was evaluated using Millipell-CM Millipell 24-well Millipore Membrane Insert culture chambers (0.4 μm, Millipore Corp., Belford, MA, USA). This system permits the exchange of metabolites in the medium but results in a physical separation between the antagonist and the pathogen. Suspensions containing the inoculum of the PsJN strain (10^6^ CFU ml^−1^) were distributed in the wells of culture plates (1 ml per well). Inserts were placed in the wells and suspensions of *B. cinerea* (10^4^ conidia ml^−1^) were dispensed into the inserts (500 μl per cylinder). Then, the plates were incubated at 20°C for 24 h in the dark. The control cultures contained *B. cinerea* alone in phosphate buffered saline (PBS). After incubation, the plates were observed under an inverted microscope. *Paraburkholderia phytofirmans* strain PsJN (initial DO_600 nm_ = 0.01) and the fungus Bc630 (10^4^ conidia ml^−1^) were grown separately or in co-culture at 22°C in a minimum liquid medium M9 (without carbon source) with agitation of 180 rpm. The optical density at 600 nm was measured and observations under the fluorescence microscope were carried out.

### Dual assays

For the mycelial growth assay, PDA plates were inoculated with 4 droplets (10 μl) of an overnight culture of PsJN and placed overnight at 28°C. Then a 5 mm plug of *B. cinerea* was placed on PDA plates with or without PsJN. The plates were incubated at 20°C for 7 days.

For analysis of volatile compounds (VOCs) on fungus development, a combination of an agar solution of “tomato medium” (tomato juice: distilled water (1:4), 15 g L^−1^ agar, pH 5.5; fungus) and King B (KB; bacteria) media were used for two-compartment plates. Twenty microliters of an overnight culture of PsJN was spread on KB compartment. Plates were placed overnight at 28°C. Then a 5-mm plug of *B. cinerea* was placed on tomato medium compartment with or without PsJN on KB. The plates were incubated at 20°C for 15 days.

### Chemotaxis

The chemotaxis between PsJN-*gfp* and *Botrytis* was tested by using a quantitative capillary assay (Supplementary Figure S1). The study of bacterial chemotaxis was realized in Petri dishes, with 5 cm long capillaries made from Pasteur pipettes (closed end). The open end of the capillary is soaked in the Eppendorf tube containing the fungal mycelium or the corresponding buffer (control). This end is then deposited in the 200 μl of bacterial solution (10^6^ CFU ml^−1^) in a corner of the Petri dish (three capillaries were deposited per Petri dish). The Petri dish containing 3 capillaries was then stored at 28°C for 45 min. After incubation, the capillaries were wiped with a 70% alcohol-wicking tissue to remove the bacteria present on the surface of the capillaries. Each capillary is then placed in an Eppendorf tube containing 1 ml of PBS. Each Eppendorf tube was then stored at 4°C for 24 h before plating serial dilutions on KB_kan_ to count colonies of PsJN-*gfp*.

### Protection assays

#### Root inoculation

Root systems of fruiting cuttings (BBCH 57; Meier, 2001) were dipped during 1 min in a bacterial inoculum (10^9^ CFU ml^−1^). The cuttings were then replaced in hermetic plastic filled with a mixture of non sterile mix, soil/perlite/sand (4/1/1) in a culture chamber (photoperiod 14 h/10 h, day/night and temperature of 20°C). When the inflorescences reached the stage BBCH 64, they were infected with *B. cinerea*. The flowers were sprayed with a conidia suspension (10^3^ conidia ml^−1^, about 1 ml per inflorescence). The cuttings were placed in moisture-saturated hermetic plastic boxes. Samples and observations using a 3D microscope (Keyence VHX-3000) and epifluorescence were performed 24 and 72 hpi with the pathogen.

#### Inflorescence spraying

Fruiting cuttings with a rooting system and fully developed inflorescences were inoculated with *P. phytofirmans* PsJN. One milliliter of a bacterial inoculum (10^8^ CFU ml^−1^) was sprayed directly on inflorescences at the flowering stage 62–63 of the BBCH scale (20–30% of flowers are open). The cuttings were then placed in hermetic plastic boxes in a culture chamber (photoperiod 14 h/10 h, day/night and temperature of 20°C). After 24 h, the inflorescences (BBCH 64) were infected with *B. cinerea*. The flowers were sprayed with a conidia suspension (10^3^ conidia ml^−1^, approximately 1 ml per inflorescence). The cuttings were placed in moisture-saturated hermetic plastic boxes. Samples and observations using a 3D microscope and epifluorescence were performed 24 and 72 hpi with the pathogen.

### Analysis of rhizosphere and endophytic colonization by PsJN

Four-week-old cuttings (4 developed leaves) were bacterized with the PsJN strain at the time of transplantation into individual pots. Bacterization was carried out at the root level by dipping the roots in a bacterial solution at 10^9^ CFU ml^−1^ for 1 min. One, two, and three weeks after inoculation, colonization tests were performed. Rhizoplane colonization was performed with 1 g of root which was vortexed with 1 ml of PBS for 1 min. Then the homogenate was diluted, and the dilutions were spread on King B agar plates (supplemented with 100 μg ml^−1^ kanamycin and 100 μg ml^−1^ cycloheximide). For endophytic colonization, the same root was disinfected for 6 min in 0.5% commercial bleach and 0.01% tween 20, followed by 4 rinses of approximately 1 min in sterile water. The samples were then ground in 1 ml of PBS. The homogenate was serially diluted and spread on King B_kan_ agar plates. Bacterial colonies were counted after 3 days of incubation at 28°C. The results obtained correspond to three independent biological replicates.

### DNA extraction and *in planta* quantification of *Botrytis cinerea* and PsJN

Genomic DNA extraction was performed according to Calderón-Cortés et al. (2010). One hundred milligrams of Pinot noir flowers were transferred to 2 ml microfuge tubes. Then a volume of 1.5 ml of extraction buffer [20 mM EDTA pH 8.0, 100 mM Tris–HCL, pH 7.5, 1.4 ml NaCl, 2% CTAB, 4% PVP-40, β-mercaptoethanol (2%)] was added to each tube. The tubes were then shaken and incubated at 60°C for 30 min. Then 2 μl of RNAase (1 mg ml^−1^) was added to each tube before being incubated at 37°C for 15 min. A volume of chloroform/isoamyl alcohol (24:1) was added, the tubes were mixed by inversion and centrifuged (for 15 min, at 3,000 rpm, room temperature). The upper aqueous portion was transferred to a new 2 ml tube and 2 volumes of cold 95% ethanol were added. After incubation at −20°C, the tubes were centrifuged (for 15 min, at 12,000 rpm, room temperature) to form a pellet containing genomic DNA. After removal of the supernatant, the pellet was resuspended in 40 μl of ultrapure water. Detection of *B. cinerea* and PsJN was performed by real time PCR and PCR, respectively, using primers described in Table 1. For real time PCR, normalization was performed using one grapevine housekeeping gene *VvEF1α* as previously described (Miotto-Vilanova et al., 2016).

**Table 1:** Primers used in this study.

Gene	Sens	Anti sens
VvEF1α	AACCAAAATATCCGGAGTAAAAGAGA	ACTGGGTGCTTGATAGGC
Actin (Bc)	CCGTGCTCCAGAAGCTTTGT	GTGGATACCACCGCTCTCAAG
GFP (Pp)	ACATCATGGCAGACAAAC	AAAGGGCAGATTGTGTG

## Results

### Lack of protection against *Botrytis cinerea* on grapevine flowers after root-inoculation with PsJN

**Figure 1:**
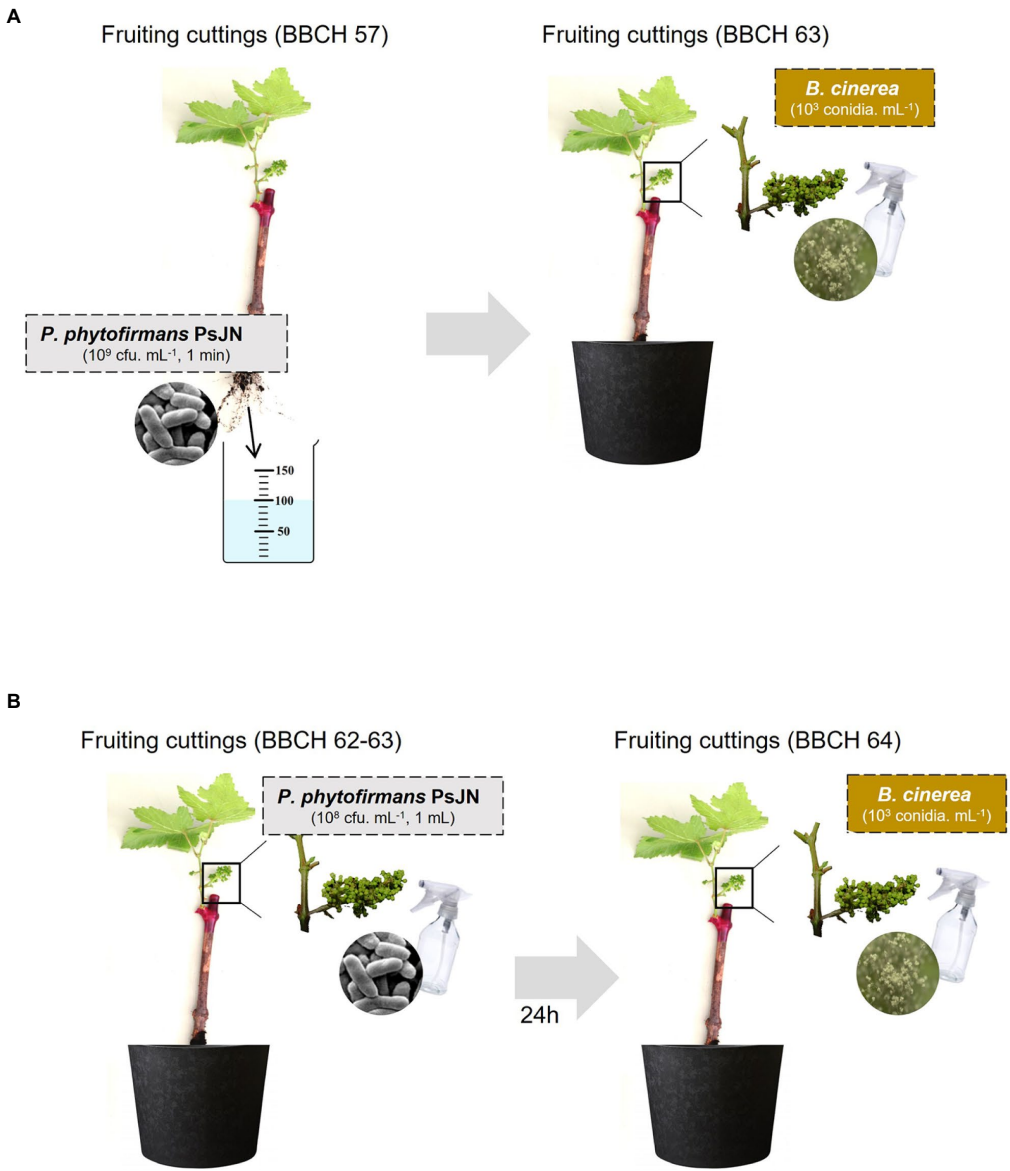
Modes of *Paraburkholderia phytofirmans* PsJN inoculation (**A**: by root, **B**: by spraying) and *Botrytis cinerea* infection used in this study.

**Figure 2:**
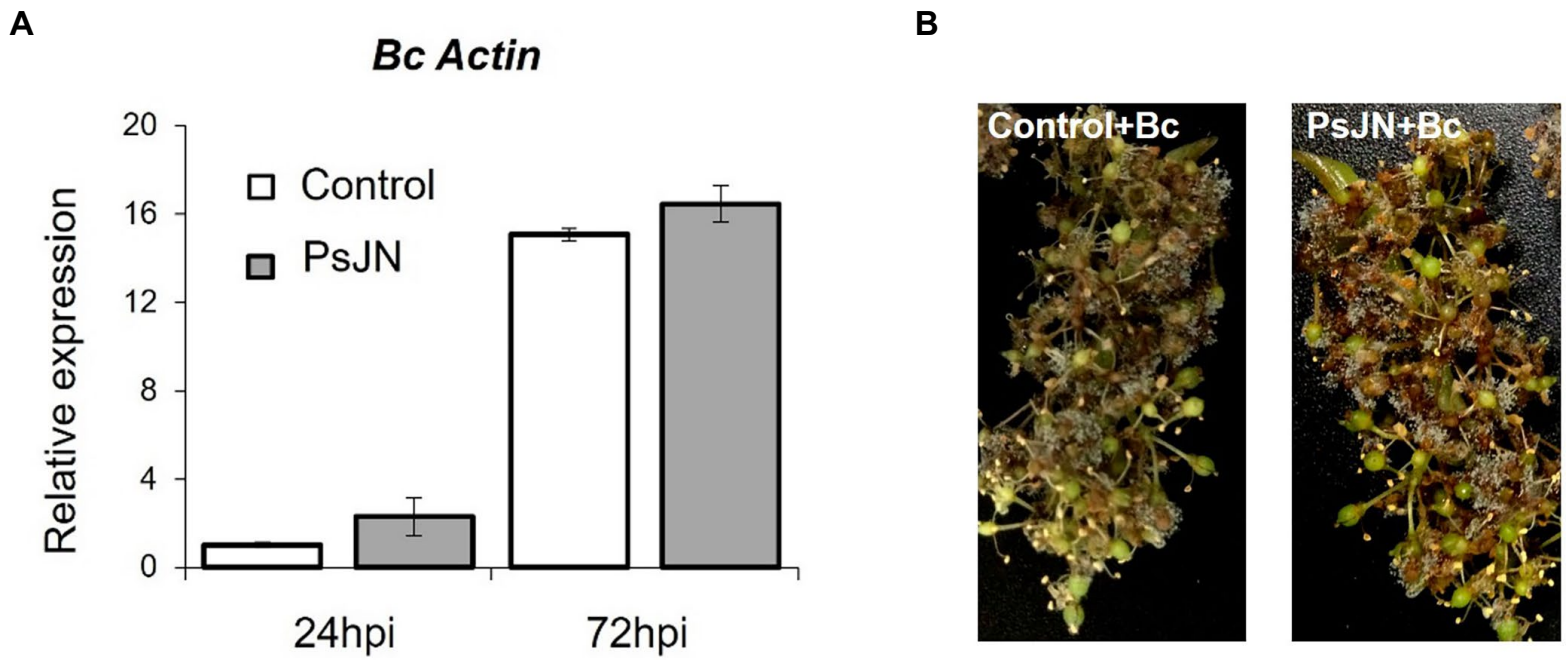
Resistance induced by *P. phytofirmans* PsJN against *B. cinerea*. **(A)** Detection of *Botrytis in planta* by qPCR using primers targeting the Actin encoding gene, at 24 and 72 hpi. **(B)** Symptoms developed on inflorescences 72 hpi with *B. cinerea* bacterized or not with PsJN.

Infection with *B. cinerea* was realized on inflorescences after root-inoculation by the beneficial bacterium (Figure 1A). In these conditions, no significant protection against *B. cinerea* was observed in the presence of PsJN (Figure 2). In parallel, the colonization profiles of PsJN on grapevine roots were established. The results showed that both colonization of the rhizoplane and internal tissues tend to decrease between one and 3 weeks after inoculation (Supplementary Figure S2), and PsJN was not detected in inflorescences (data not shown).

### Interaction between PsJN and *Botrytis cinerea*

**Figure 3:**
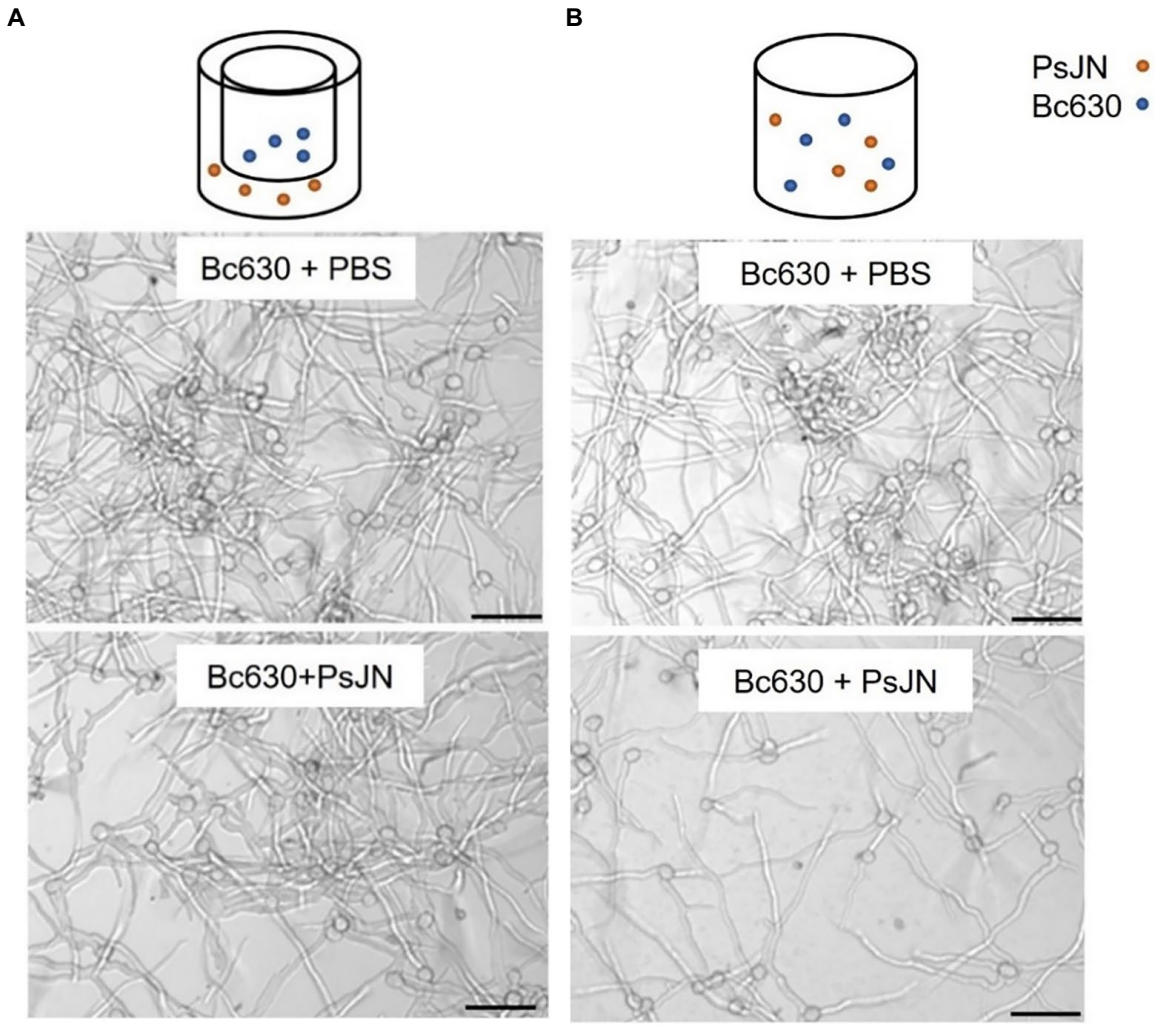
Direct antifungal effect of *P. phytofirmans* PsJN on *B. cinerea* spore germination with **(A)** or without **(B)** contact between the microorganisms. Observations were realized under inverted microscope 24 h after co-inoculation of PsJN (10^6^ CFU ml^−1^) and *B. cinerea* (5 × 10^4^ conidies ml^−1^). Scale bars = 50 μm.

As we reported that a simultaneous application of PsJN and *B. cinerea* on *in vitro*-plantlets leaves could protect grapevine against *B. cinerea* through a direct antimicrobial effect on spore germination (Miotto-Vilanova et al., 2016), we deeper investigated herein the *in vitro* antifungal effect of PsJN (10^6^ CFU ml^−1^) against *B. cinerea* strain 630 (5 × 10^4^ conidia ml^−1^). Using Millipell-CM Millipell 24-well Millipore Membrane Insert culture chambers, outcomes demonstrated that the inhibition of *Botrytis* spore germination by PsJN requires a physical contact between the two microorganisms (Figure 3).

**Figure 4:**
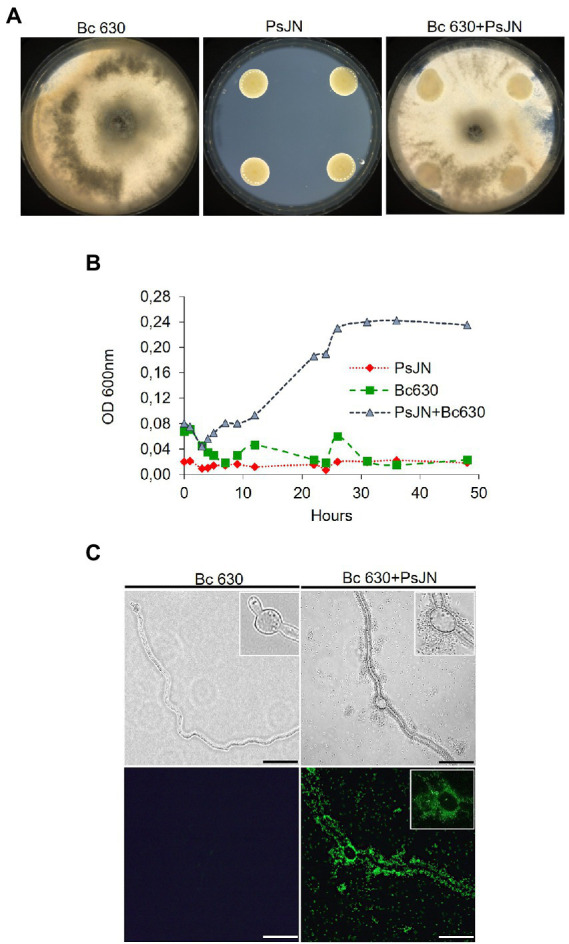
Co-culture of *P. phytofirmans* PsJN and *B. cinerea*. **(A)** Mycelial growth of *B. cinerea* with PsJN-*gfp* or not on PDA solid medium (in standard 90 mm Petri dishes). **(B)** Growth curves of PsJN and *B. cinerea* cultivated separately or both in minimal liquid medium. **(C)** Observations of *B. cinerea* mycelium 24 hpi with PsJN-*gfp* or not in liquid minimal medium, under fluorescence microscope. Scale bars = 100 μm.

No significant inhibition of mycelial growth of *B. cinerea* on solid medium (PDA) in the presence of the bacterium was observed (Figure 4A). The ability of PsJN to inhibit the mycelial growth *via* the production of VOCs showed no effect of the bacterium (Supplementary Figure S3). Finally, the growth of PsJN, with or without the fungus, was measured in liquid cultures. The results indicated that in a minimum medium, PsJN or *Botrytis* alone cannot grow but, in co-culture, the bacterium could use the fungus as a source of nutrients for its own growth (Figure 4B). To complete, microscopic observations of the co-culture were realized and showed that *P. phytofirmans* PsJN-*gfp* forms a biofilm around fungal hyphae 24 h after the onset of co-culture (Figure 4C).

**Figure 5:**
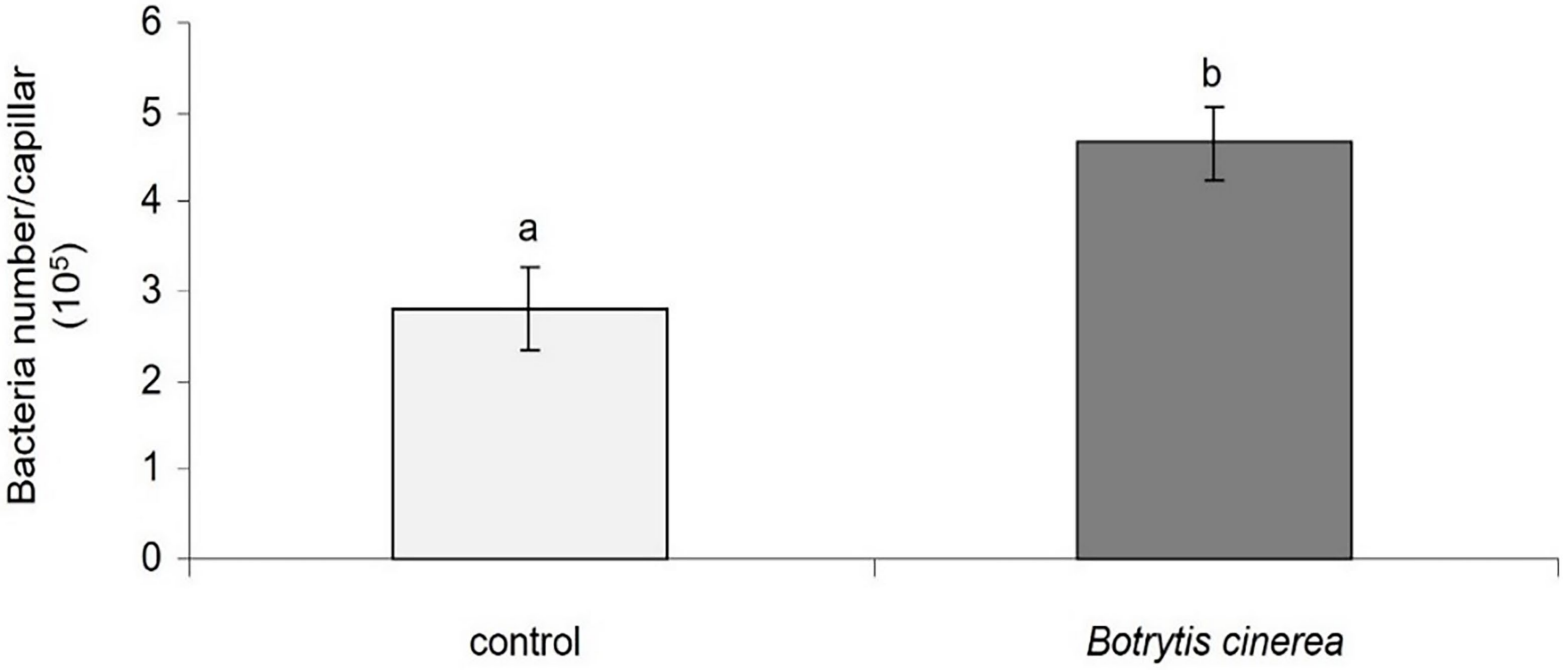
Chemotaxis of *P. phytofirmans* PsJN toward *B. cinerea*. Data presented are means +/− SD from three biological repetitions. Different letters above each bar indicate a significant difference (*p* < 0.05) as determined by Student’s *t* test.

Also, the chemotaxis of PsJN toward *B. cinerea* strain 630 by a quantitative capillary assay showed that *B. cinerea* attracts by positive chemotaxis the beneficial bacterium PsJN (Figure 5).

### Resistance against *Botrytis cinerea* induced by direct spraying of *Paraburkholderia phytofirmans* PsJN on grapevine inflorescences

**Figure 6:**
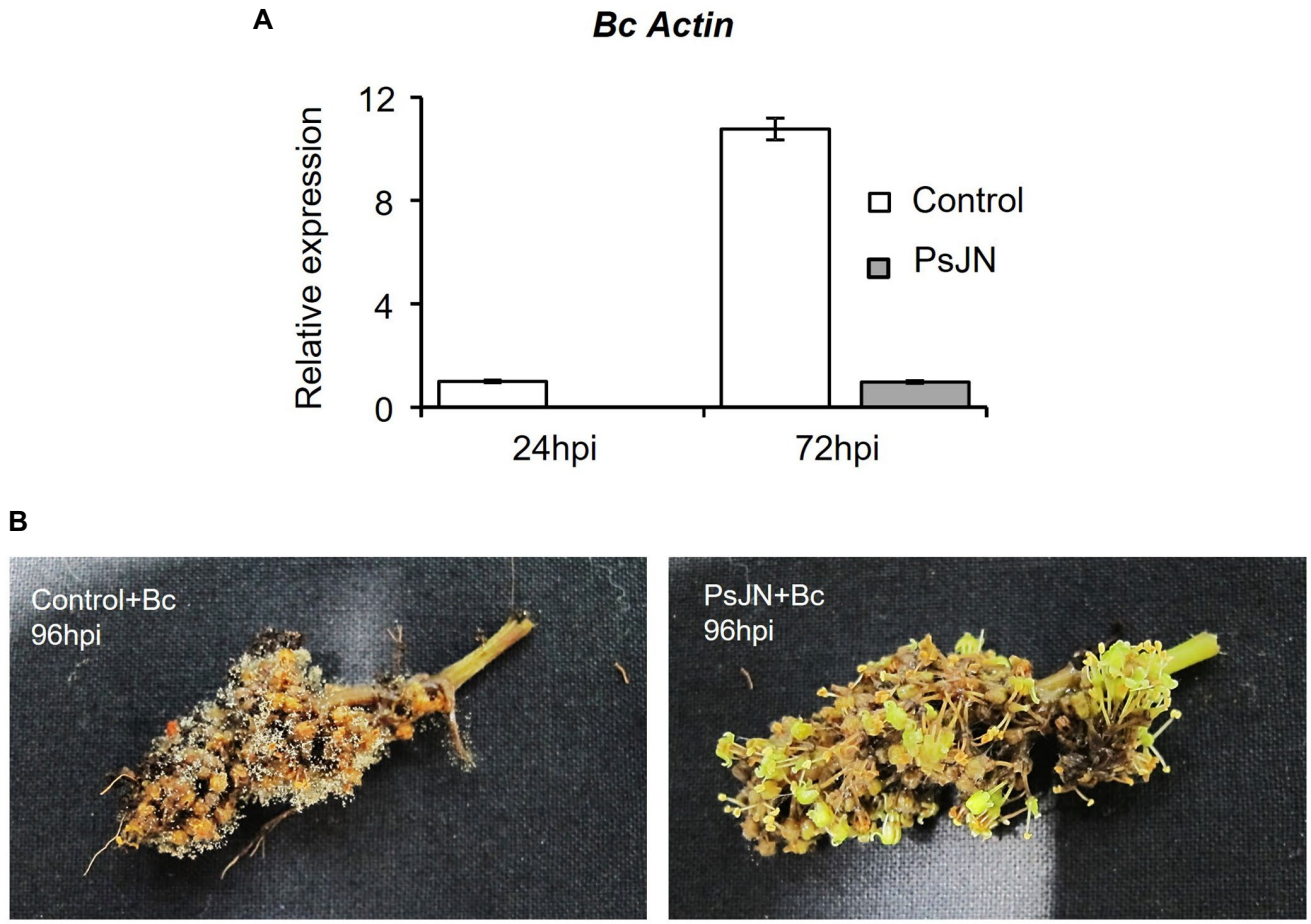
Resistance induced by *P. phytofirmans* PsJN against *Botrytis cinerea*. **(A)** Detection of *B. cinerea in planta* by qPCR using primers targeting the Actin encoding gene, at 24 and 72 hpi. **(B)** Symptoms developed on inflorescences bacterized or not at 96 hpi with *B. cinerea*.

**Figure 7:**
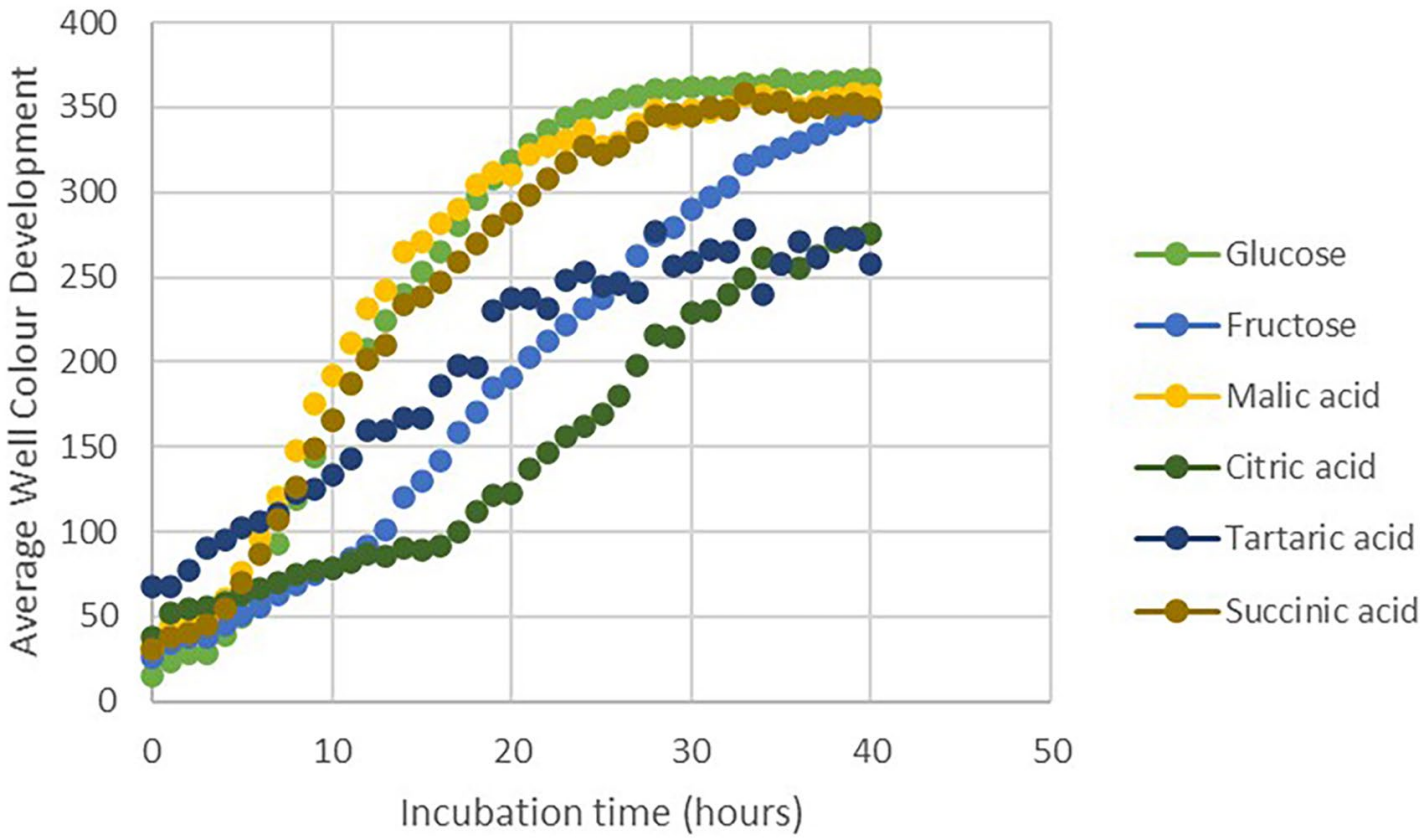
Utilization of different carbon sources by PsJN using the Biolog (PM1 and PM2) microplate method.

Considering the antifungal effect of *P. phytofirmans* PsJN against *B. cinerea*, a direct spray of *P. phytofirmans* PsJN on grapevine inflorescences was performed at the stage 62–63 of the BBCH scale (Figure 1B), just before fully flowering; considered as the more susceptible phenological stage to *Botrytis* infection (Keller et al., 2003; van Kan et al., 2014). Briefly, PsJN was sprayed directly on grapevine inflorescence (1 ml of 10^8^ CFU ml^−1^) and grapevine cuttings were placed in hermetical boxes in order to maintain a high humidity. Twenty four hours after bacterial inoculation, plants were infected with *B. cinerea* strain 630 (1 ml of 10^3^ conidia ml^−1^) and placed in hermetical boxes. *In planta* quantification of the *B. cinerea* Actin gene (*Bc Actin*), realized by real time PCR, showed a significant reduction in the level of the *Bc Actin* transcript in bacterized plants compared to the control ones at 24 and 72 hpi (Figure 6A), indicating a protective effect conferred by PsJN. In parallel, the strain PsJN-*gfp* was still detected by PCR 72 h after *B. cinerea* challenge (data not shown). We observed also that PsJN showed high metabolic activity on glucose, fructose and malic-, tartaric-, citric- and succinic acids that are major sugars and organic acids of grapevine (Figure 7), which could give it a competitive advantage on inflorescences. Four days after the infection with *B. cinerea*, the development of the pathogen was delayed in bacterized inflorescences compared to those treated only with PBS (Figure 6B).

### Co-localization of both beneficial and pathogenic microorganisms on grapevine receptacle and stigma

**Figure 8:**
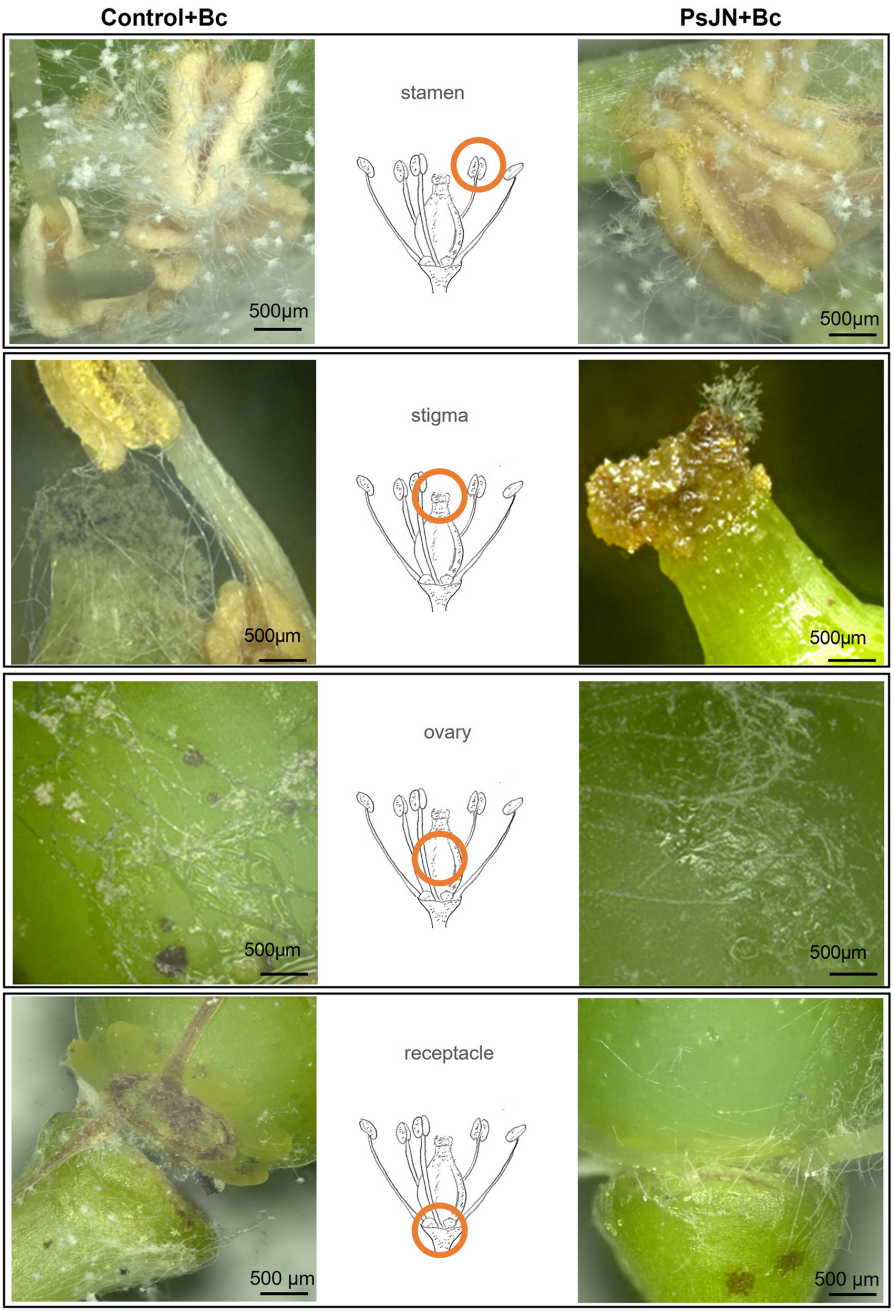
*Botrytis cinerea* development on grapevine inflorescences inoculated or not with PsJN. The inflorescences were sprayed with PsJN (10^8^ CFU ml^−1^) or with PBS as control. Twenty-four hours after, *B. cinerea* was sprayed (1 ml of a 10^3^ conidia ml^−1^). 3D-microscopic observations were realized 72 hpi by the fungus on different parts of the flowers. Scale bars = 500 μm.

3D-microscopic observations were conducted on the different parts of infected flowers in control and bacterized plants: stamens, stigma, ovary, and floral receptacles (Figure 8). Results showed that following the artificial infection by spraying, *B. cinerea* can colonize all parts of the flowers. The beneficial bacterium PsJN seemed to have no protective effect on stamens and ovaries (Figure 8). On the other hand, the development of *B. cinerea* on stigma and floral receptacles is more important in control plants than in bacterized ones. The necrosis caused by the pathogenic fungus in the receptacle area leads to abscission and the fall of the flowers in the non-bacterial inflorescences (Figure 8).

**Figure 9:**
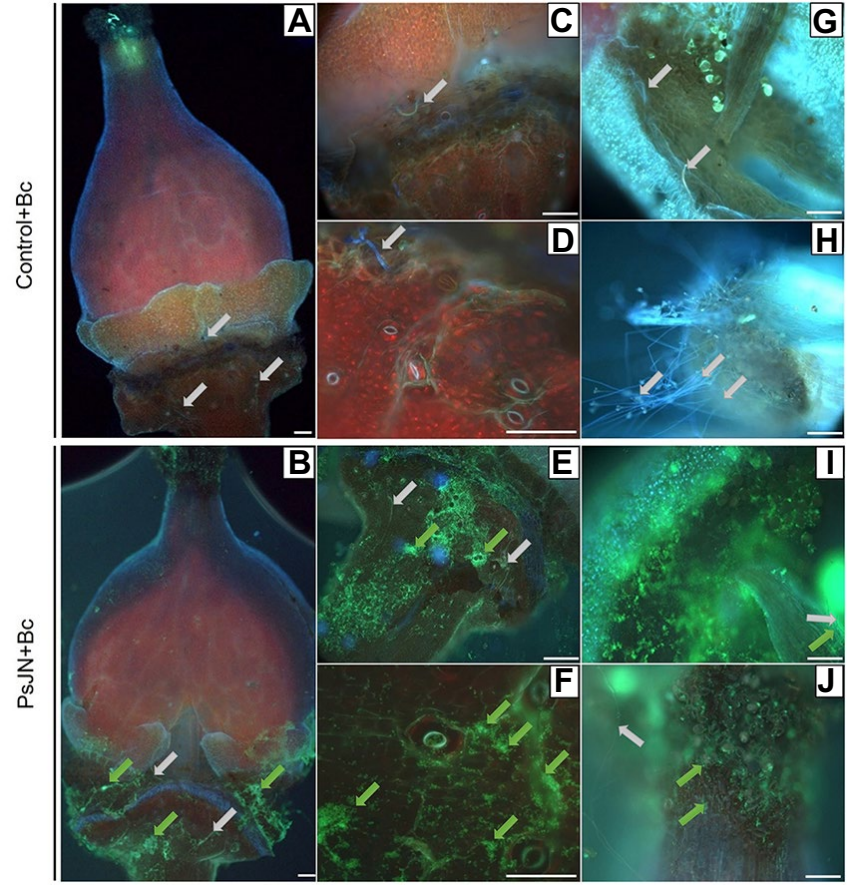
*Botrytis cinerea* development and *Paraburkholderia phytofirmans*-*gfp* localization on grapevine inflorescences. The inflorescences were sprayed with PsJN-*gfp* (10^8^ CFU ml^−1^) or with PBS as control. Twenty-four hours after, *B. cinerea* was sprayed (1 ml of a 10^3^ conidia ml^−1^). Microscopic observations were realized 72 hpi by the fungus on flowers **(A,B)**, receptacle **(C–E)**, stomata **(D–F)**, stamen **(G–I)** and stigmate **(H–J)**. Scale bars = 100 μm. Green arrows: PsJN-*gfp*, grey arrows: *B. cinerea.*

In parallel, using the fluorescence microscope, observations on the inoculated flowers showed that the PsJN-*gfp* bacterium is mainly found on the stamens (Figure 9I), the stigma (Figure 9J) and on the receptacle (Figures 9A,B,C,E). The bacterium was also detected in the stomata located in the flower receptacle (Figures 9D,F). According to our experiments, *B. cinerea* can develop on the receptacle. We can notice that the bacterium was present in large quantities at the receptacle, which may explain the less marked infection by *B. cinerea*. This co-localization of the beneficial bacterium and the pathogen on stigma and receptacles might explain the protective effect induced by PsJN against *B. cinerea via* a direct antimicrobial effect.

## Discussion

BCAs can be used as biofungicides in a range of 10^5^–10^9^ CFU ml^−1^ (Pertot et al., 2017; Abbey et al., 2019; Poveda et al., 2020) and diverse mechanisms implied in bacterial-fungi interactions are described: antibiosis, signaling and chemotaxis, physico-chemical changes after adhesion and protein secretion (Deveau et al., 2018). Miotto-Vilanova et al. (2016) highlighted that a simultaneous co-inoculation of PsJN and *B. cinerea* on *in vitro-*plantlets leaves leads to a protection against the pathogenic fungus *via* a direct antifungal effect. To explore this property, we deeper investigated the bacterium-fungus interaction. We thus observed an inhibition of spore germination, in accordance with previous data (Miotto-Vilanova et al., 2016). However, we showed for the first time that this inhibition effect is effective only when there was a direct contact between PsJN and *B. cinerea* but not when a membrane filter, which permits medium nutrients and metabolite interchange, separated the beneficial bacterium and the fungus. It is known that antagonism exerted by bacteria belonging to *Burkholderia/Paraburkholderia* species is largely related to the production of several antifungal compounds (Partida-Martinez and Hertweck, 2007; Vial et al., 2007; Schmidt et al., 2009) that can inhibit a broad range of phytopathogenic fungi (Quan et al., 2006; Kilani-Feki et al., 2011; Groenhagen et al., 2013). PsJN is known to produce secondary metabolites (Esmaeel et al., 2018); however, PsJN did not inhibit the mycelial growth of *B. cinerea* on PDA medium (Figure 4A), which may indicate that this strain does not produce antibiotic substances against the fungus. Finally, the exact mechanism by which PsJN inhibits *Botrytis* spore germination is not yet clear but makes PsJN a good candidate to protect against grey mold disease since it has been proposed that initial spore density regulates the amplitude of attack and defense (Veloso and van Kan, 2018).

The importance of chemotaxis and direct physical contact in bacterial-fungal interactions is well described (Nazir et al., 2010; Frey-Klett et al., 2011; Haq et al., 2016). We performed a chemotaxis test and showed that PsJN is positively attracted by the pathogenic fungus *B. cinerea* (Figure 5) and can form a biofilm around the fungal hyphae in liquid co-culture (Figure 4C). These latter results are in agreement with observations made *in planta* where PsJN was detected at the leaf surface, surrounding the fungal mycelium only in botrytized-leaves (Miotto-Vilanova et al., 2016). VOCs emitted by plant associated bacteria are described as promising antifungal agents (Bailly and Weisskopf, 2017; Bruisson et al., 2019). The potential of PsJN to inhibit the growth of *B. cinerea* through the emission of VOCs was also tested and we showed that PsJN cannot limit the fungal growth *via* this mechanism (Supplementary Figure S3) but interestingly, we observed a difference between control and PsJN conditions 15 days after inoculation. Indeed, *B. cinerea* can invade the whole box in the control condition, but its development remains restricted to its compartment in the presence of PsJN (Supplementary Figure S3), thus confirming the effectiveness of direct physical contact between the two microorganisms.

Many non-pathogenic microorganisms suppress the growth of plant pathogens through competition for nutrients. After *B. cinerea* infection, the PCR data showed the presence of the PsJN on grapevine inflorescences. The survival of PsJN on grapevine inflorescences and its ability to use nutrients are key elements since concentration and survival are the most essential factors that influence the outcome of biocontrol system (Jeger et al., 2009). We demonstrated also in this study the ability of PsJN to use major sugars (glucose, fructose) and organic acids (malic-, tartaric-, citric- and succinic acids) of grapevine (Figure 7). A high rhizosphere competence is a prerequisite for biocontrol activity when BCAs are applied on root systems (Barret et al., 2011; Ghirardi et al., 2012; Schreiter et al., 2018). In this study, we showed that after root-inoculation of grapevine fruiting cuttings, the survival of PsJN on rhizosphere and internal root tissues tend to decrease over the time (Supplementary Figure S2). These observations may be correlated with the fact that PsJN does not confer an effective and significant resistance of grapevine inflorescences against *B. cinerea* (Figure 2). These results are in accordance with previous data demonstrating that low level of endophytic PsJN subpopulations were detected in inflorescences of grapevine plants after root-inoculation and only in 10–60% of bacterized fruiting cuttings (Compant et al., 2008).

As the correlation between *in vitro* inhibition and *in planta* control of infection development is not always observed (Haidar et al., 2016), and based on the *in vitro* antifungal effect of PsJN against *B. cinerea*, we tested a direct spraying on grapevine inflorescences, and showed that PsJN is able, *via* this mode of inoculation, to significantly delay the development of *B. cinerea.* The bacterization by a direct spray of inflorescences with *P. phytofirmans* PsJN was carried out at BBCH 62–63 stage, when 20–30% of the flowers were open, before full flowering, which is considered the most susceptible phenological stage to *B. cinerea* infection (Keller et al., 2003; van Kan, 2006). After artificial spray infection, we observed that *B. cinerea* colonized the entire flower zone. The fungal grows primarily on the caps, sepals and stamens. Observations showed a higher development of *B. cinerea* in the caps of the control plants than in the plants bacterized with PsJN, indicating that the bacterium inhibited the development of the fungus in this area. These results are interesting since previous study showed that flower caps and other plant debris (flowers and stamens) could be colonized by the fungus which remains dormant in the developing cluster (Elmer and Michailides, 2007). At 72 hpi, the fungus was present all over the receptacle area and particularly in the space between the receptacle and the ovary. The presence of a large space between the receptacle and the ovary, after the cap has fallen off, can indeed serve as a receiving channel or channel for conidia. Conidial germ tubes located in the open space above the receptacle can penetrate the inner part of the flower by developing between the cells of the ovary and the receptacle (Figure 6). Furthermore, we showed that PsJN-*gfp* bacteria are mainly found on the stamens, the stigma and especially in the receptacle region. The receptacle region is the main entry point for the fungus (Viret et al., 2004) and the stigma infection route was reported (McClellan, 1973) from field experiments as the infection route responsible for early *B. cinerea* rot. However, it was shown that inoculation of the stigma and ovary did not generally lead to infection, although conidia germinated on these organs (Viret et al., 2004). The receptacle area thus appears to be a central entry point for the fungus. This region is important since the attack of this part by the fungus can cause abscission and flower drop. Our study shows that the presence of *P. phytofirmans* strain PsJN in this area can inhibit and delay the development of *B. cinerea*.

In conclusion, we showed that PsJN can be used, *via* its direct effect on spore germination, as a biofungicide to control gray mold disease in grapevine.

## Data availability statement

The original contributions presented in the study are included in the article/Supplementary material, further inquiries can be directed to the corresponding author.

## Author contributions

All authors listed have made a substantial, direct, and intellectual contribution to the work and approved it for publication.

## Conflict of interest

The authors declare that the research was conducted in the absence of any commercial or financial relationships that could be construed as a potential conflict of interest.

## Publisher’s note

All claims expressed in this article are solely those of the authors and do not necessarily represent those of their affiliated organizations, or those of the publisher, the editors and the reviewers. Any product that may be evaluated in this article, or claim that may be made by its manufacturer, is not guaranteed or endorsed by the publisher.
