# Supplementary material for: Paraburkholderia phytofirmans PsJN delays Botrytis cinerea development on grapevine inflorescences
Source: Front Microbiol. 2022 Oct 20;13:1030982. doi: 10.3389/fmicb.2022.1030982 (PMC9630468; doi:10.3389/fmicb.2022.1030982)
Supplement: Supplementary file 1 [file Data_Sheet_1.PDF]

Supplementary data

Paraburkholderia phytofirmans PsJN delays *Botrytis cinerea* development on grapevine inflorescences

Lidiane Carla Vilanova Miotto[1#], Marine Rondeau[1#*], Mathilde Robineau[1], Jean François Guise[1], Céline Lavire[2], Ludovic Vial[2], Florence Fontaine[1], Christophe Clément[1], Cédric Jacquard[1], Qassim Esmaeel[1], Ait Barka Essaïd[1##], Lisa Sanchez[1##]

Supplementary figure S1: Workflow for chemotaxis analyses.

Supplementary figure S2: Grapevine colonization by *P. phytofirmans* PsJN.

Supplementary figure S3: Volatile-mediated growth inhibition of *B. cinerea* by PsJN, at 6 and 15 days

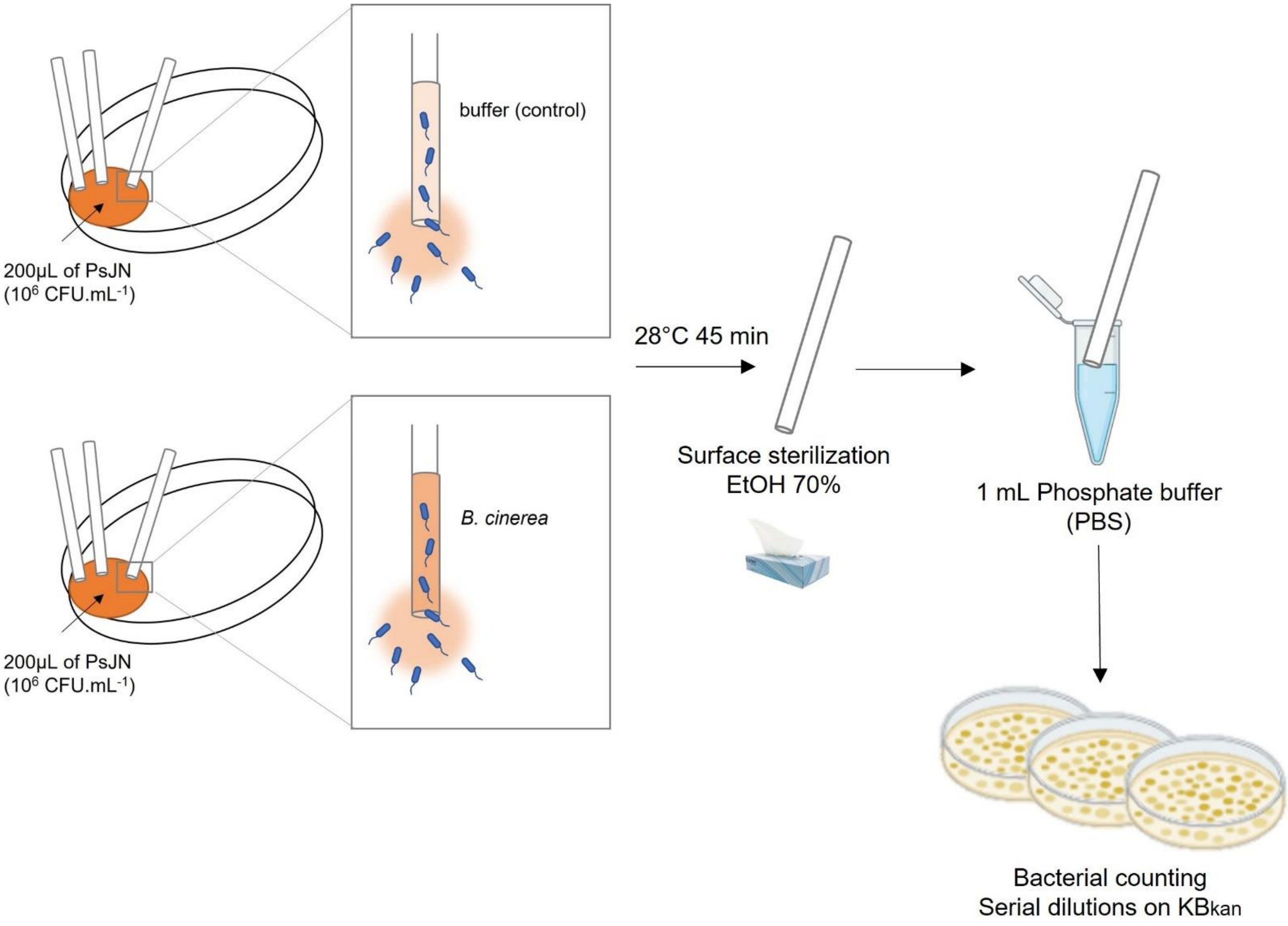

Supplementary figure S1: Workflow for chemotaxis analyses.

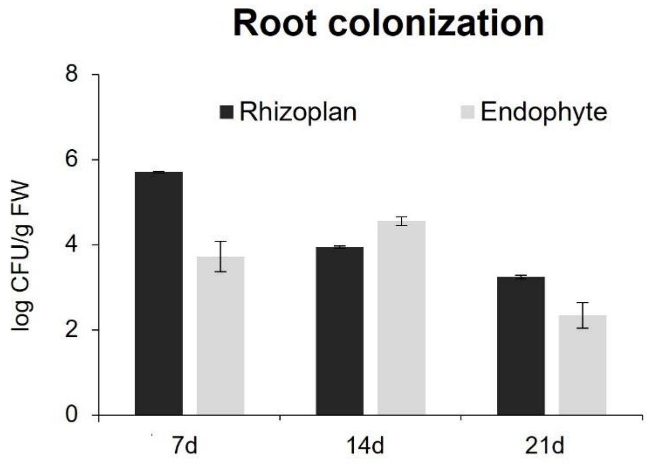

Supplementary figure S2: Grapevine colonization by *P. phytofirmans* PsJN.

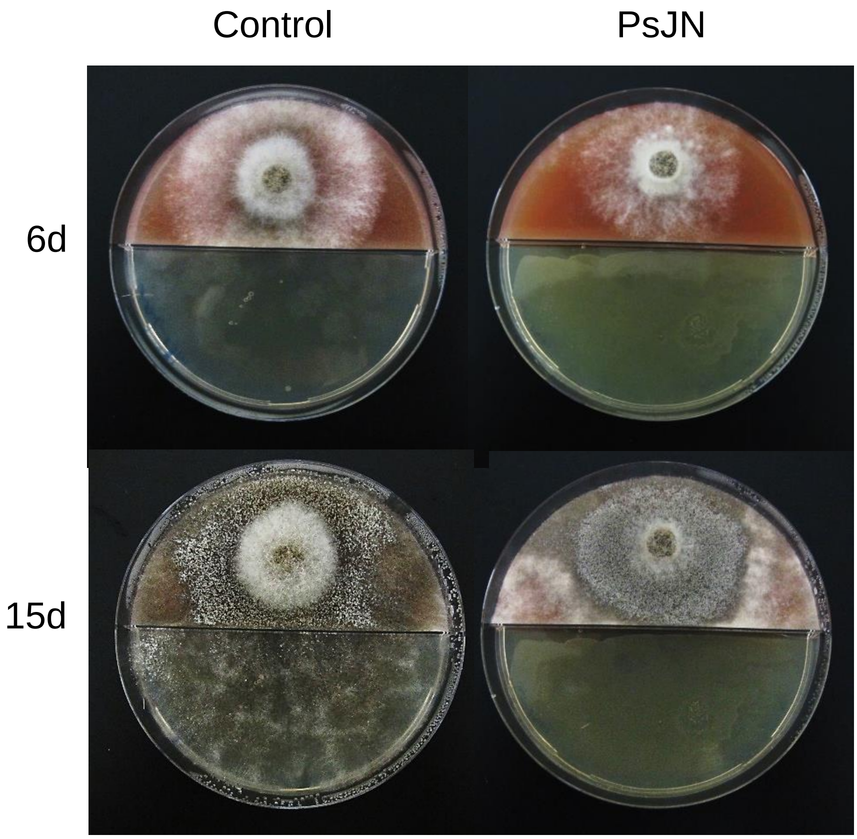

Supplementary figure S3: Volatile-mediated growth inhibition of *B. cinerea* by PsJN, at 6 and 15 days
